# Development of Innovative Biocomposites with Collagen, Keratin and Hydroxyapatite for Bone Tissue Engineering

**DOI:** 10.3390/biomimetics9070428

**Published:** 2024-07-15

**Authors:** Florin Popescu, Irina Titorencu, Madalina Albu Kaya, Florin Miculescu, Raluca Tutuianu, Alina Elena Coman, Elena Danila, Minodora Maria Marin, Diana-Larisa Ancuta, Cristin Coman, Adrian Barbilian

**Affiliations:** 1 Department of Orthopedics and Traumatology, Faculty of Medicine, University of Medicine and Pharmacy “Carol Davila”, 8 Eroii Sanitari Bvd., 050474 Bucharest, Romania; popescu_florin_md@yahoo.com (F.P.); adrian.barbilian@yahoo.com (A.B.); 2 Institute of Cellular Biology and Pathology ‘’Nicolae Simionescu’’, 8 B. P. Hasdeu Street, District 5, 050568 Bucharest, Romania; irina.titorencu@gmail.com (I.T.); ralu.tutuianu@gmail.com (R.T.); 3 INCDTP—Division Leather and Footwear Research Institute, 93 Ion Minulescu Str., 031215 Bucharest, Romania; elena.danila23@yahoo.com; 4 Department of Metallic Materials Science, Physical Metallurgy, National University of Science and Technology Politehnica Bucharest, 313 Independenței Spl., 060042 Bucharest, Romania; f_miculescu@yahoo.com; 5 Advanced Polymer Materials Group, National University of Science and Technology Politehnica Bucharest, 1-7 Polizu Street, 011061 Bucharest, Romania; minodora.marin@ymail.com; 6 “Cantacuzino” National Medical-Military Institute for Research and Development, 103 Independenței Spl., 050096 Bucharest, Romania; diana.larisa.ancuta@gmail.com (D.-L.A.); comancristin@yahoo.com (C.C.)

**Keywords:** biomimetic composites, keratin, collagen, hydroxyapatite, biocompatibility, mesenchymal stem cells, regenerative medicine, scaffold biodegradation

## Abstract

This study follows the process for the development of an innovative biomimetic composite derived from bovine collagen with keratin, with hydroxyapatite being hybridized into its architecture, and it builds a comprehensive evaluation of the composite’s characteristics. The novel biomimetic materials are tailored with special traits to be achieved for the repair of osteochondral defects (OCDs). The purpose of the present research is to create a reliable effective alternative to existing bone graft materials while leveraging the intrinsic properties of the components for enhanced osteoinduction and integration. The composites were characterized based on their morphological properties, including water absorption, through scanning electron microscopy (SEM), and their structural properties were characterized by Fourier-Transform Infrared Spectroscopy (FTIR). Biological performance was assessed in vitro using human bone marrow mesenchymal stem cells (BMSCs), focusing on cytotoxicity, cell viability, and the ability to support cell colonization with forthcoming results. This in vivo study illustrates the real potential that this class of novel composites exhibits in regard to bone and cartilage tissue engineering and encourages further exploration and development for future clinical applications.

## 1. Introduction

Millions of people worldwide suffer from bone disease each year as a result of trauma, tumors, fractures, or malformations that put a tremendous amount of pressure on all medical systems [1,2]. Sadly, some patients lose their lives because of inappropriate therapy or unsuitable or improper bone substitutes.

Osteochondral defects (OCDs) or lesions (OCLs) are localized injuries of the articular cartilage that also affect the nearby subchondral cancellous bone and subchondral bone plate. Even though OCDs can occur in any joint in the human body, osteochondral abnormalities are most frequently observed in the knee. Chondral refers to cartilage, and osteo indicates bone [3,4]. A technique to treat OCDs is performing a transplant procedure, bone grafting. Currently, different categories of transplant exist, such as repairing this deficiency with tiny plugs of bone and cartilage from other parts of the knee. The bones can be transplanted from the same patient (autograft) or from other persons (allograft) or a synthetic bone can be used [5]. Thousands of surgical procedures are performed daily to treat OCDs damaged by accidents or diseases. When applying allograft or autograft transplant procedures, the side effects are the possibility of bacterial infection, immunogenic response, and disease transmission from the graft donor for allograft, need of second surgical procedure, limited availability or donor site pain for autografts. In order to eliminate these side effects, synthetic bone materials are used to create scaffolds around which natural bone grows [6].

Biomimetics is a multidisciplinary field that seeks to mimic natural processes and structures to develop innovative materials and technologies. In the realm of tissue engineering, biomimetic materials hold great promise for creating scaffolds that closely resemble the extracellular matrix (ECM), providing an ideal microenvironment for cell growth, tissue regeneration, and integration with host tissues. This study focuses on the development and characterization of a novel biomimetic composite material composed of collagen, keratin, and hydroxyapatite, aiming to harness the structural and biochemical cues of bone and cartilage tissues for enhanced tissue regeneration. Presently, an extensive selection of biomaterials is employed for developing scaffolds for bone grafting. This paper is specifically focused on evaluating the potential of keratin and collagen of animal origin to be used as base ingredients for scaffold manufacturing. Several studies regarding biomaterials have been developed for bone grafting and cartilage tissue engineering, with a wide range of proteins, including collagen, albumin, gelatin, fibroin, and keratin, being explored for the development of naturally derived biomaterials to treat OCDs.

Composites based on collagen and hydroxyapatite are maybe the most used bone biomaterials, having the ability to simulate and substitute skeletal bones [7]. For example, a biomimetic scaffold made of hydroxyapatite and collagen has been properly created using human adipose stem cells, and the scaffold’s biophysical characteristics and diverse biological functions were examined. In a mouse model of posterolateral lumbar spinal fusion, this biomimetic microchannel scaffold greatly enhanced new bone formation and blood vessel ingrowth in vivo, and it also showed tremendous potential for osteogenic activities in vitro [8]. In another study, hybrid hydrogels composed of poultry collagen with UV–riboflavin crosslinking revealed low osteo-conductivity, biocompatibility, and biodegradability.

Compared with collagen, collagen–nano-keratin, or collagen–apatite–nano-keratin formulations, the collagen–apatite group exhibited improved functional stability, highlighting potential for development as an osteo-promoting membrane [9]. Because collagen is the primary structural protein present in the extracellular matrix of connective tissues, has exceptional properties, and is biocompatible, it is intensively used in bone reconstruction. In a successful in vivo investigation, the osteo-regenerative potential of collagen/chitosan/hydroxyapatite scaffolds, obtained by two distinct hydroxyapatite manufacturing methods, was examined in defects formed in rats’ healthy tibial bone as well as in fragile bone caused by gonadal hormone deficit (ovariectomy) [10].

Keratin is a versatile biopolymer that has been employed for the creation of fibrous composites. It may also be transformed into gels, films, nanoparticles, and microparticles by applying the appropriate technologies of their preparation. Applications in biomedical sciences and regenerative medicine have gained advantages for their resistance against enzymatic degradation and distinct biocompatibility [11]. In the body, keratin affords protection and support. It is composed of a wide variety of proteins, such as enzymes extracted from animal tissues, keratin-associated proteins (KFAPs), and multiple types of keratins. Moreover, isolated keratins can self-assemble into structures that govern the identity and behavior of individual cells. Keratin is extracted from hair, toenails, nails, horns, beaks, claws, and feathers from chickens. Wool is another important source [12]. Keratin-based materials are attractive candidates for revolutionizing the biomaterial market due to biocompatibility, biodegradability, mechanical durability, and natural abundance [11,12,13]. Upon research and characterization, an osteo-inductive biocomposite scaffold composed of human hair keratin, jellyfish collagen, and nano-sized spherical hydroxyapatite generated from eggshells was created and has the potential to be implemented in bone tissue engineering [14]. Using a straightforward freeze-drying method, a novel composite scaffold was successfully developed by mixing hydroxypropyl methylcellulose and hydroxyapatite with keratin produced from sheep wool. The produced scaffold’s maximal compressive strength was found to be within the range of human trabecular bone, indicating a highly porous and linked network, leading the composite to be an adequate bone substitute [15]. Other authors applied novel approaches to synthesize boron and silicon cryogels with incorporated collagen/hair keratin developed for hard tissue engineering applications [16]. Another study reported an antibacterial wound dressing based on keratin (K)–sodium alginate (A) loaded with green synthesized zinc oxide nanoparticles (ZnO NPs) to be used for skin regeneration [17]. Bone scaffolds based on polymers have many disadvantages such as a low degradation rate, low hydrophilicity, low fragility, and low cell compatibility. Due to the disulfide, hydrogen, and ionic bonds in keratin, biomaterials based on polymers and keratin can have improved tensile strength and elastic moduli. Moreover, research has also demonstrated the antibacterial activity, cell adhesion, and hydrophilicity of keratin as a biomaterial for biomedical applications [18].

Wu et al. showed that keratin had an important role as a crosslinking agent for collagen fiber films inducing highly improved mechanical properties and enhanced thermal stability (a 31.95% increase in elongation at break was achieved for the films prepared by crosslinking collagen fiber with 50% keratin) [19]. Recently a construct for osteogenic induction in bone tissue engineering was built based on keratin, polyhydroxy butyrate, and alumina nanowire with successful results [20]. The keratin–montmorillonite nanocomposite hydrogels enhanced osteogenic differentiation and dramatically stimulated bone regeneration in vivo, being a promising candidate in bone tissue engineering [21].

Biomaterials used for bone grafting are continuously and rapidly developing. In this paper, we propose an innovative biomaterial with bovine collagen, keratin, and hydroxyapatite for bone restoration, combining the properties of each component. As far as we know, these materials have not been used together until now. The composites developed were characterized by water absorption, enzymatic degradation, scanning electron microscopy, biocompatibility with cells, and animal tests. The results proved that the developed biomaterials can be successfully used for bone grafting.

## 2. Materials and Methods

### 2.1. Preparation of Biocomposites

Type I collagen as fibrillar gel with an initial concentration of 2.39% and acidic pH was obtained from calf skin according to our technology as we previously described [22]. Briefly, collagen derma was treated with organic acid and then the fat was removed; it was washed and treated with alkali to remove the non-collagenous protein and washed and solubilized in acid again. After all the derma was treated, precipitation followed. Composite gels based on collagen gel, keratin from Merck (Darmstadt, Germany), and bovine derived-hydroxyapatite [23] were prepared according to the composition given in Table 1. Hydroxyapatite was obtained by thermally processing deproteinized bovine bone pieces at 1000 °C, followed by milling at ceramic particles sizes of 100–200 µm. Different concentrations (0.5, 1.0 and 1.5%) of keratin were added to samples F2–F4 and F6–F8, and the samples F5–F8 consisted of the same ratio of collagen/hydroxyapatite = 1:1. All the composite gels consisted of a final concentration of collagen of 1%, and they were crosslinked with 0.5% glutaraldehyde from Merck (Hohenbrunn, Germany).

**Figure 1:**
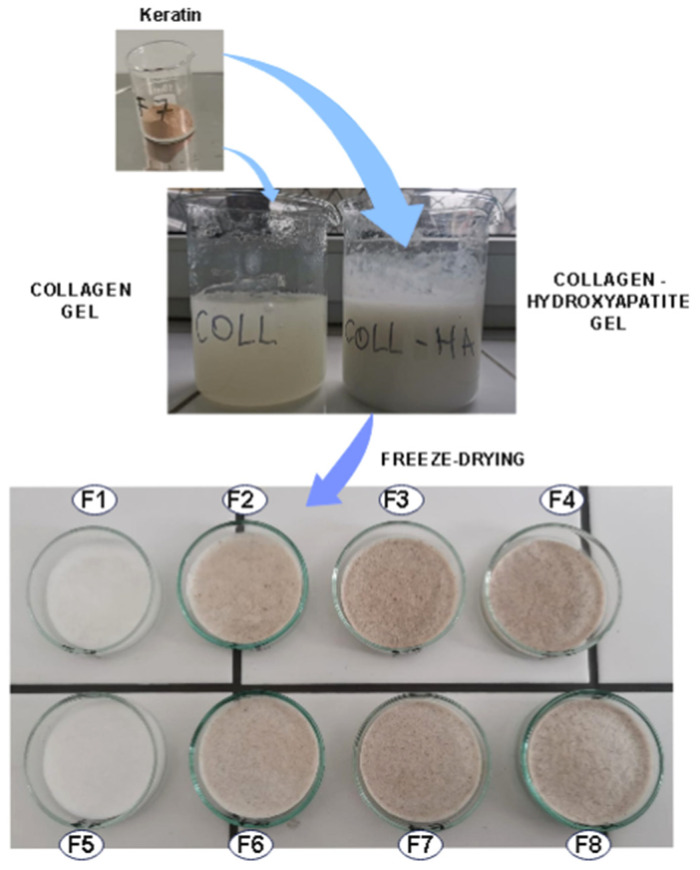
Schematic preparation of collagen biocomposites in form of sponges.

The composite gels were poured in a glass Petri dish and freeze-dried for 48 h using the Martin Christ 24 Delta LSC freeze-dryer (Martin Christ Gefriertrocknungsanlagen GmbH, Osterode am Harz, Germany) [24] to obtain spongious forms (Figure 1). The gels already poured in Petri dishes were pre-frozen at −40 °C for about 8 h and then the main freeze-drying started at −40 °C and 0.01 bar, reaching 10 °C in 20 h, 20 °C in 8 h, 30 °C in another 8 h, and final freeze-drying for 4 h at 0.001 bar at 30 °C. After 48 h we, obtained the sponge-like samples.

The biocomposite sponges were then gamma irradiated with 25 kgy to be sterile for in vivo tests. The gamma irradiation was done by subcontractor (IRASM Department of IFIN-HH Romania) using a Co-60 gamma irradiator (Institute of isotopes—Budapest, Hungary), an industrial, versatile, panoramic irradiator.

### 2.2. Water Uptake of Biocomposites

The water uptake capacity of the biocomposite sponges was determined by the weight method [25], by weighing the sponges before and after immersion in water. A piece of sponge of about 1 cm^3^ was weighed at a reference time and then it was placed in 3 mL of water at room temperature (about 23 °C). At predetermined periods of time (1, 2, 4 h and 1, 2, 3, 4 and 7 days), the sponges were weighed and the capacity to retain water was calculated using the following equation (Equation (1)):

Water uptake (%) = [(Wt − Wd)/Wd] × 100(1)

where Wt represents the weight of water retained by sponges at time t and Wd is the weight of dry sponges. Data were presented as mean ± SD of three independent experiments.

### 2.3. Fourier-Transform Infrared Spectroscopy (FTIR)

FTIR measurements were performed with an attenuated total reflectance (ATR) accessory on a Vertex 70 Bruker FTIR spectrometer (Billerica, MA, USA). The ATR-FTIR mode was used to register the FTIR spectrum for each obtained formulation, with a resolution of 4 cm^−1^ in the 600–4000 cm^−1^ wavenumber range.

### 2.4. Scanning Electron Microscopy of Biocomposites

The morphology of the biocomposite sponges was observed using the scanning electron microscope Quatro^TM^ (Thermo Fisher Scientific, Hillsboro, OH, USA).

### 2.5. Cell Culture of MSCs on Biocomposites

Human mesenchymal stem cells (MSCs) were isolated after informed consent with the approval of the Institutional Ethical Committee (180/27 September 2018), in accordance with the most recent version of the Helsinki declaration of the World Medical Association (Ethical Principles for Medical Research Involving Human Subjects, October 2008).

Patients who were under surgical treatment for osteoarthritis complications were included in this study.

Human bone marrow-derived MSCs were isolated using a modified protocol established and already published by our group [26].

Briefly, BM aspirate (c. 4 mL) was obtained by puncture of the postero-superior spine of the iliac crest.

The cells were collected in cold DMEM containing 4.5 g/L glucose (Sigma Aldrich, St. Louis, MO, USA) supplemented with 40 UI/mL heparin, 300 UI/mL penicillin, 300 µg/mL streptomycin, and 150 µg/mL neomycin (all for Sigma Aldrich, St. Louis, MO, USA), and sedimented by centrifugation at 160 g for 10 min at 4 °C. The nucleated cells were separated on a Histopaque 1077 (Sigma Aldrich, St. Louis, MO, USA). The cells were placed on top of the Histopaque and, after centrifugation at 650 g for 10 min (4 °C), one white band was obtained. The cells from the band were recovered, washed twice with DMEM supplemented with 15% fetal bovine serum (Gibco BRL, Gaithersburg, MD, USA), seeded at a density of 10^5^/cm^2^, and grown in DMEM 4.5 g/L glucose supplemented with 15% fetal bovine serum (FBS), 1% non-essential amino acids, 300 UI/mL penicillin, 300 mg/mL streptomycin, and 150 mg/ mL neomycin, at 37 °C and 5% CO_2_. The culture medium was changed after 24 h and then twice a week. All the manipulations were performed according to good manufacturing practice (GMP) criteria and good laboratory practice (GLP). The cells were characterized following the indications of the International Society for Cellular Transplantation.

The cells were characterized as previously described [25]. The cells were plated at 10^4^ cell/cm^2^ and grown in DMEM 1 g/L glucose supplemented with 1% non-essential amino acids (Sigma Aldrich, St. Louis, MO, USA), 10% fetal bovine serum (FBS), and 1% penicillin, streptomycin, and neomycin, at 37 °C and 5% CO_2_.

### 2.6. Viability Assessment

The effect of the Ker:COL:HA scaffolds on the viability of human bone marrow mesenchymal stem cells (BMSCs) was evaluated using the XTT assay. Prior to cell seeding, the scaffolds were sterilized with 70% ethanol overnight at RT and then washed with sterile water. The sterilized samples were placed in DMEM supplemented with 10% FBS and placed in the incubator at 37 °C in 5% CO_2_. Cells at a density of 150,000/cm^2^ were cultured on the scaffolds placed in a 96-well plate (37 °C, 5% CO_2_) for 7 days. Cell proliferation was evaluated using the XTT assay viability/proliferation kit (Thermo Scientific, Waltham, MA, USA), according to the manufacturer’s instructions, and the results were reported as a percentage of the positive control (collagen matrices—F1). Data were presented as the mean ± SD of two independent experiments. Two-group analysis was carried out by Student t test. Probability values (*p*) < 0.05 were considered significant (*, *p* < 0.05, **, *p* < 0.01, ***, *p* < 0.001, ns, not significant).

### 2.7. Assessment of Biocomposite Capacity to Support Colonization of MSCs

Following the viability tests, the samples that supported MSC growth were selected: F1, F5, and F6. MSCs were seeded at a density of 150,000/cm^2^ on top of the scaffolds placed in a 48-well plate. After 4 h, the samples were transferred to new wells to further cultivate only the BMSCs that adhered to the matrix. The medium was changed every two days, and on day seven post-seeding, the scaffolds were fixed in 4% PFA (paraformaldehyde) (Sigma Aldrich, St. Louis, MO, USA), overnight, at 4 °C, followed by inclusion in Shandon Cryomatrix and cryosectioning.

To assess the cell colonization capacity of the scaffolds, we performed an eosin-Hoechst staining as previously described [27]: the sections were washed in PBS, followed by 2 min in eosin and differentiation in 70% ethanol, distilled water for 3 min, and Hoechst solution (1 mg/mL) for 10 min. After a final wash and mounting with glycerol, the samples were visualized using a Zeiss Observer D1 fluorescence microscope.

### 2.8. Immunofluorescence (IF) Staining

The cryosections were rinsed for 15 min with PBS, blocked in 10% BSA for 15 min, and then incubated for 1 h with primary antibodies directed against vimentin (1:150; mouse monoclonal anti-vimentin (Sigma Alrich, St. Louis, MO, USA)) and fibronectin (1:500, rabbit polyclonal antibody (Thermo Fisher Scientific, Waltham, MA, USA)). In the next step, the cells were incubated for 1 h with secondary antibodies: 1:1000 Alexa-Fluo 568 goat antimouse IgG (Thermo Fisher Scientific, Waltham, MA, USA) and 1:1000 Alexa-Fluo 488 goat antimouse IgG (Thermo Fisher Scientific, Waltham, MA, USA). After three washing steps, the cryosections were mounted with Fluoroshield with DAPI (Sigma Alrich, St. Louis, MO, USA) and visualized using a Zeiss Observer D1 fluorescence microscope. Digital fluorescence images were captured after the optimal exposure time was determined based on control samples to exclude background staining.

### 2.9. Animal Studies

The animal study protocol was approved by the Ethics Committee of “Cantacuzino” National Medical-Military Institute for Research and Development Bucharest (CI) and by the competent authority (Directorate of Veterinary Health and Food Protection, Bucharest)—Project authorization no. 15 of 17 May 2023.

The sample size was set at 10/group using a simplified calculation formula [28], and to reduce the number of animals, both hind limbs were used for testing, one for the test material and the congener that served as control. Therefore, for this study, thirty 20-week-old male Wistar strain non-specific pathogen (SPF) rats were used. The rats’ weight ranged between the 250 and 300 g intervals at baseline. The animals were supplied by the CI, Băneasa Animal Facility (BAF), and maintained in cages (Tecniplast, Italy) with autoclaved bedding. Cage changes were scheduled to be performed once a week. Rats were distributed in study groups of a maximum of five animals per cage and were exposed to a 12 h/12 h (day/night) light/darkness cycle in a ventilated room with a controlled temperature of 20–24 °C and free access to sources of food and filtered water.

The CI’s Preclinical Testing Unit and Experimental Medicine and Translational Research Platform, where the animal experiments were set to take place, had an Environmental Enrichment Programme. Human targets were determined using a clinical examination based on the ARRIVE criteria, with clinical symptoms graded according to severity. Animal health status was evaluated daily by a veterinarian specialist. Animals had a period of acclimatization to the new environment prior to beginning the experiments. In the cage, rats were individually identified by colored permanent marker according to the CI identification procedure.

Surgical Procedure: the surgical intervention that was designed involved preoperative preparation, anesthesia, local infiltrations, incisions, the creation of a cavity in the femur, insertion of the test device, suture, and postoperative care including antibiotics and analgesics.

Prior to surgery, the rats underwent a fasting period to diminish anesthesia risks. The surgical area, at the rats’ lower limbs, was shaved and disinfected with a topical solution of Iodine 3%.

For the anesthesia, a combination of Ketamine (Vetased, Farmavet, Romania, 75 mg/kg) and Medetomidine (Dorbene Vet, Altius, Romania, 0.5 mg/kg) was used, thus obtaining the surgical comfort needed and a painless, less stressful procedure for the animals. Anesthesia depth was regularly assessed by reflex testing, and the eyes were covered with an ophthalmic ointment.

An osteochondral bone defect in the shape of a tunnel for the insertion of biomaterials was created through a surgical approach that exposes the trochlear groove, following the study protocol described by Ancuta et al. [29]. Under saline solution cooling in order to minimize thermal damage to the tissue, a drilling machine with 1 mm and 2 mm drill bits was used to allow a gradual enlargement to a desired 2 mm wide tunnel.

The development of the femoral defects involved both limbs. According to the experimental design, the left limb received the test material treatment corresponding to the group it belonged to, while the right limb with the defect remaining untreated was considered the control.

The biomaterial application involved creating a cavity in the femur through a surgical approach that exposed the trochlear groove. The cavity, designed to mimic osteochondral defects, was gradually enlarged using a drilling machine with 1 mm and 2 mm drill bits to achieve a 2 mm wide tunnel. The biomaterial was then inserted into this cavity. The exact size of the biomaterial pieces used in the study was standardized to fit the created defects, ensuring consistency across experiments. The pieces were carefully inserted into the bone defects created in the femurs of the rats. The biomaterials were applied in a hydrated state. Prior to implantation, the materials were sterilized and hydrated with saline solution to mimic physiological conditions and ensure optimal interaction with the surrounding tissue.

At the end of the surgical interventions, the animals were placed in clean cages in the experimental space, protected from noise, and received a substance (Atipamezol 0.25 mg/kg—Alzane, Altius, Romania) to quickly reverse the effect of the anesthetic. Also, postoperatively, all measures were taken to reduce the animals’ pain and prevent infections, administering antibiotics (Enrofloxacin 5 mg/kg, Farmavet, Romania) and anti-inflammatory medication (Ketoprofen 5 mg/kg, Dopharma Vet, Giroda, Timis) for five days.

## 3. Results and Discussion

### 3.1. Morphology and Structure of Biocomposites

The sponges based on collagen with/without hydroxyapatite and keratin were morphologically characterized by water uptake and SEM, while the structural changes were estimated by FT-IR.

**Figure 2:**
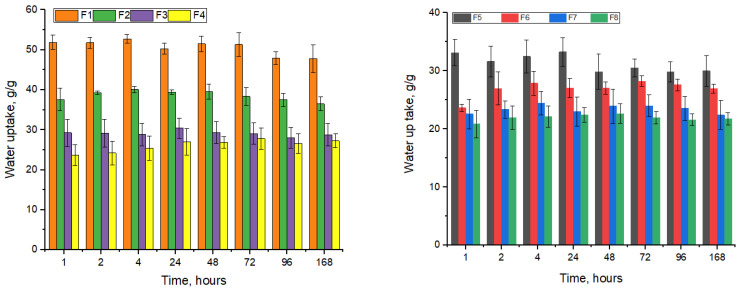
Water uptake of biocomposites.

The water uptake was estimated for one week at different intervals of time and showed that all the samples were stable as Figure 2 shows.

The samples containing only collagen absorbed about 51 g/g water, and the one with keratin, being denser, absorbed a smaller amount of water. The higher concentration of keratin resulted in a smaller amount of water uptake, as follows: F2, which contains 0.5% keratin, up-took about 38 g/g; F3, which contains 1% keratin, up-took about 29 g/g; and F4, which had the highest amount of keratin (1.5%), absorbed about 23 g/g water. The same trend can be noticed in the samples (F5 ÷ F8) which contain hydroxyapatite (HA), but the values are smaller, with the structure of the samples being denser because of the HA content. Comparing the samples with and without HA, it can be observed that F5 (with HA content) absorbed about 35% water compared with F1 (only crosslinked collagen), which up took about 51 g/g in the first 24 h. Such decreases are visible in all the samples with HA, F6, F7, and F8, which absorbed about 24, 23, and 21 g/g water.

**Figure 3:**
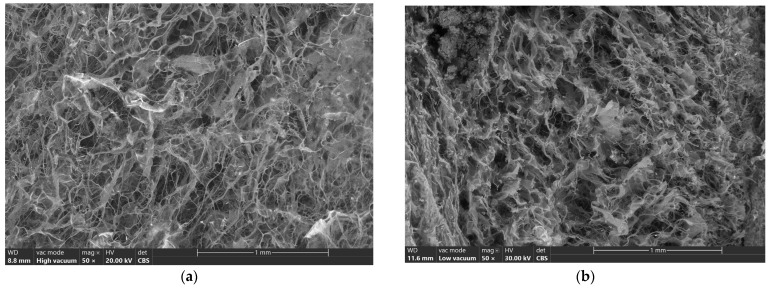

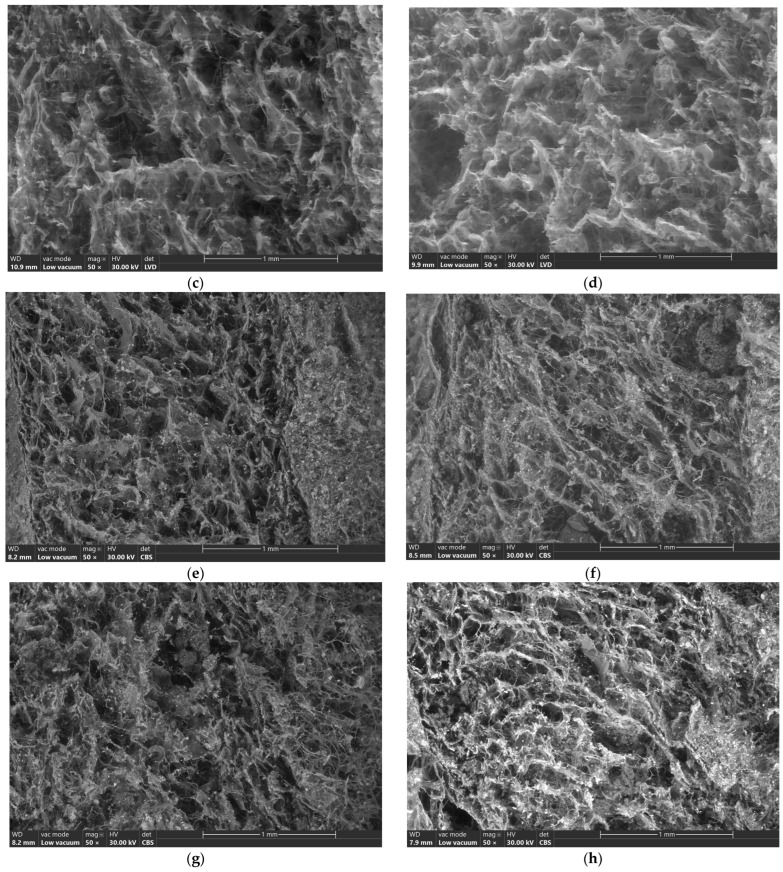

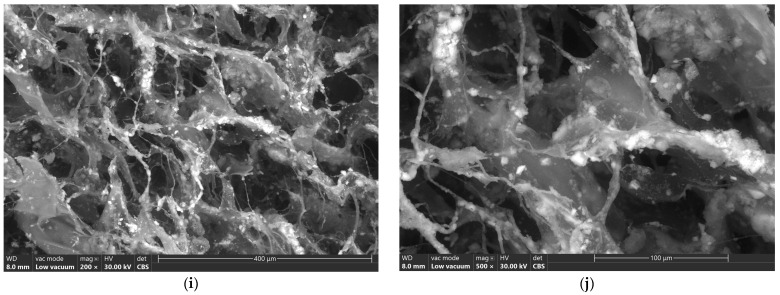
SEM images of biocomposites (×50): (**a**) F1; (**b**) F2; (**c**) F3; (**d**) F4; (**e**) F5; (**f**) F6; (**g**) F7; (**h**) F8; (**i**) F8 (×200); (**j**) F8 (×400).

These results are confirmed by the SEM images presented in Figure 3.

All samples presented a spongious structure with pore sizes between 50 and 200 µm, which is ideal for bone scaffolds [30]. The structure of the samples became denser when keratin was added for all the samples, and the HA was attached on collagen fibers in samples F5 ÷ F8. The results are in correlation with those for water absorption and show that the control sample with only collagen (F1) has bigger pores, and their sizes decrease with the amount of keratin, and the smallest ones are for samples which also contain HA (Figure 3e–h). The HA is visible on collagen fibers for all the samples with HA, as you can see, for example, in Figure 3i for sample F8 at ×200 magnification, as well as in Figure 3j.

**Figure 4:**
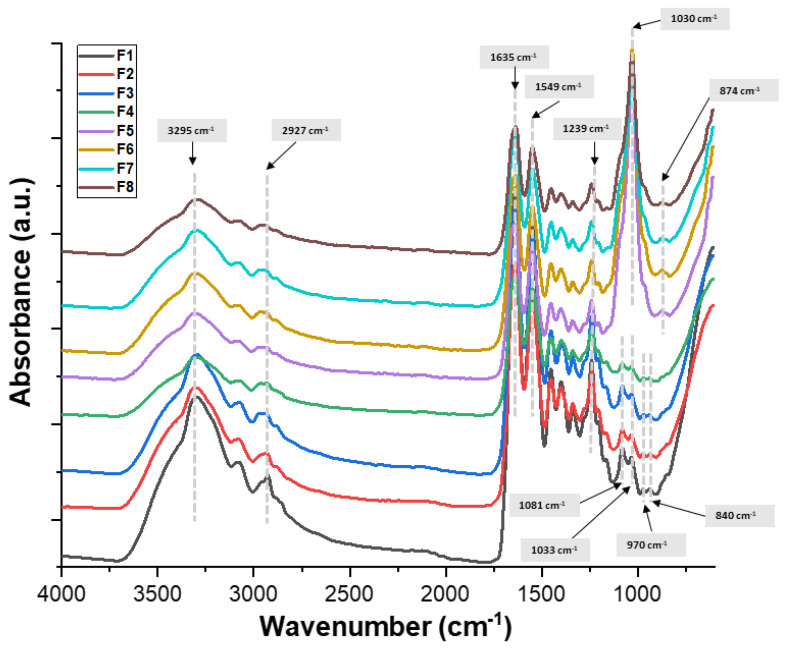
FTIR spectra of biocomposites.

The samples based on collagen, keratin, and hydroxyapatite were spectrally analyzed by FTIR spectroscopy (Figure 4). The FTIR investigations were performed on the freeze-dried samples.

The spectra of composite biomaterials made of collagen, keratin, and hydroxyapatite showed the distinctive infrared bands of each constituent. In the obtained FTIR spectra, the characteristic absorption band for peptide bonds (–CONH–) due to the presence of both proteins, keratin and collagen, can be observed [31]. Also, the amide bands in infrared spectra identify the triple-helix conformation that the collagen macromolecule displays [32]. The amide A and B bands, which are primarily connected to the NH stretching vibrations, OH groups, and CH asymmetric vibration, are responsible for the bands located at around 3295 cm^−1^ and 2927 cm^−1^ [33,34]. The stretching vibrations of the C=O groups in peptides are responsible for the amide I band at 1635 cm^−1^. The band at 1549 cm^−1^, which is attributed to amide II, is caused by CN stretching vibrations. The NH bending vibrations from amide linkages are responsible for the amide III band, which is located at 1239 cm^−1^ [35]. The presence of carbonate at 874 cm^−1^ revealed by the FTIR spectra is attributed to the hydroxyapatite’s distinctive absorption bands [35].

### 3.2. In Vitro Behavior of Biocomposites

**Figure 5:**
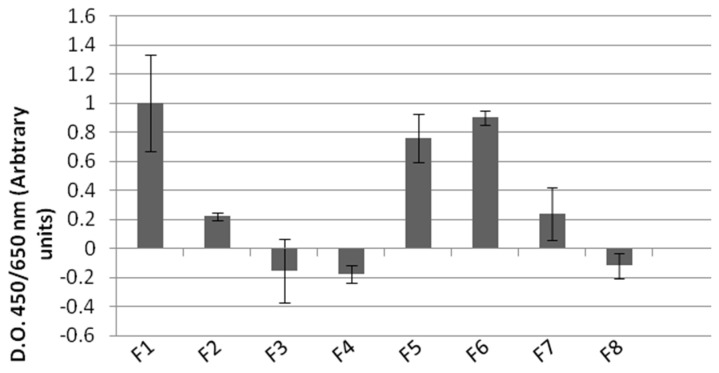
Evaluation of cytotoxic effect of the scaffolds using the XTT assay. The viability of MSCs cultured for 7 days on the specimens was assessed. Data represent mean ± SD of two independent experiments.

The cytotoxicity of the Coll:Ker:HA scaffolds was assessed by means of the XTT test using MSCs. The cells cultured on the collagen matrix had the highest viability, this viability being preserved only in the case of samples F6 and F5. In the cases of F3, F4, and F8, the test indicated an inhibition of cell growth, as seen in Figure 5.

**Figure 6:**
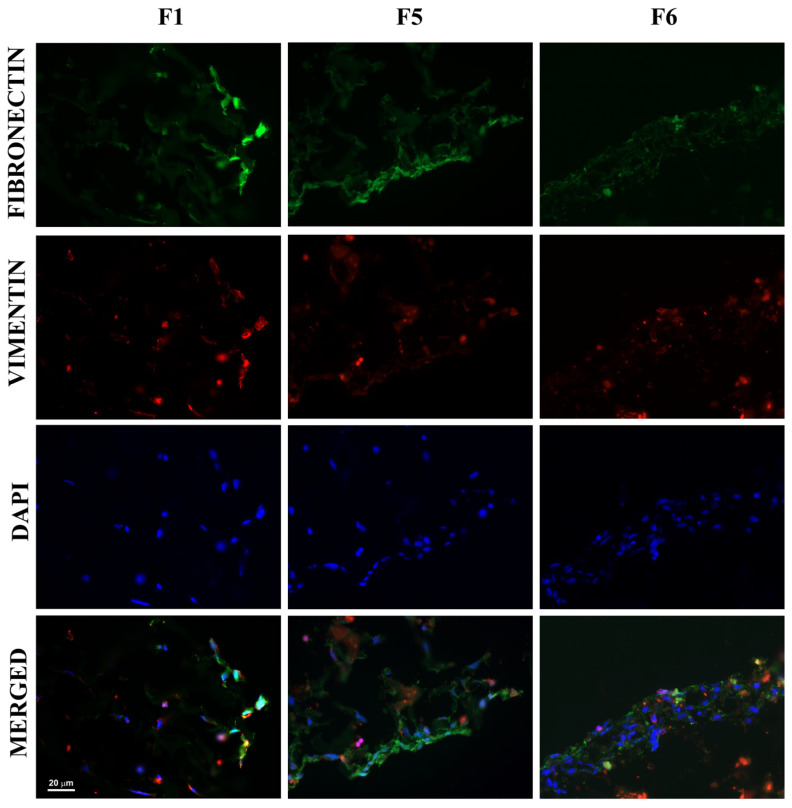
Fibronectin and vimentin expression (mesenchymal markers) by MSCs grown on Coll:Ker:HA scaffolds for 7 days.

The Coll:Ker:HA scaffolds seeded with MSCs were analyzed in respect to the production of the most characteristic ECM components. This analysis was performed after 7 days of cultivation to ensure proper cell adaptation to the 3D environment. Vimentin, a basic mesenchymal cell marker and important participant of cell sprouting and migration [36], was present in MSCs grown on Coll scaffolds and more discreetly distributed in MSCs cultured on F5 and F6—Figure 6. Fibronectin, which acts as a key player for the communication between the intra- and extracellular environment, was observed in MSCs grown on all tested scaffolds [37]—Figure 6.

**Figure 7:**
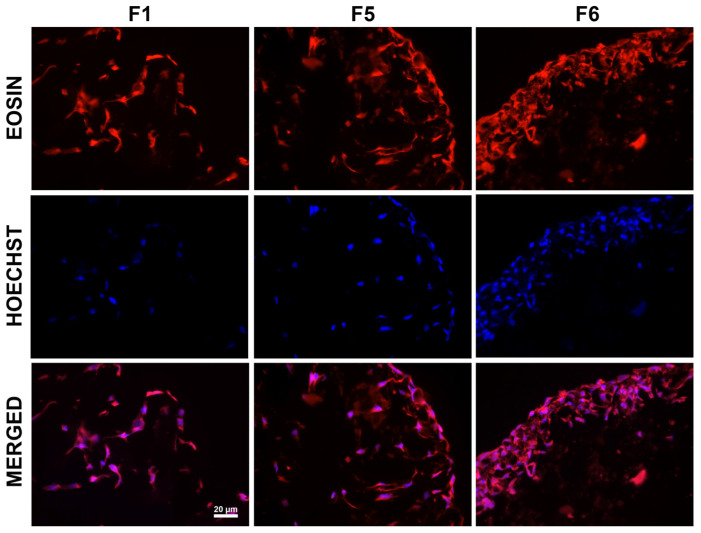
Images of eosin-Hoechst staining for the selected scaffolds seeded with MSCs, 7 days post-cultivation.

Regarding the colonization capacity of selected scaffolds, the eosin–Hoechst staining revealed a uniform distribution of MSCs within the F1 sample, while for F5 and F6, the cells mainly grew on the surface and less within the 3D structure—Figure 7. Furthermore, the viability results for these two samples, with F6 supporting a better effect on MSC proliferation, were confirmed by the thickness of the cell layer, which was higher than for F5, as seen in Figure 7.

### 3.3. In Vivo Behavior of Biocomposites on Animals

The advancement of samples F1, F2, F5, and F6 into further testing is rooted in their distinctive in vitro compositional attributes and their exhibited stability. Sample F1, noted as ctrl, solely comprising collagen, acts as a fundamental control for gauging the impact of the additional components. Sample F2 introduces keratin at a 0.5% concentration without hydroxyapatite, providing insight into keratin’s role in the composite matrix. Sample F5 explores the interaction between collagen and hydroxyapatite in a 1:1 ratio, devoid of keratin, aiming to investigate the combined potential of these materials. Sample F6, enriching the biocomposite with both 0.5% keratin and hydroxyapatite, maintains the collagen to hydroxyapatite ratio, thereby exploring the synergistic effects of these components together. These selections highlight the significance of the compositional balance and the ratios of materials in the development of biocomposites tailored for biomedical use. The progression to animal models aimed to validate the regenerative potential observed during the in vitro testing. As a consequence of the in vitro study, the samples F1, F2, F5, and F6 were found to be the best candidates for the in vivo experimental model.

**Figure 8:**
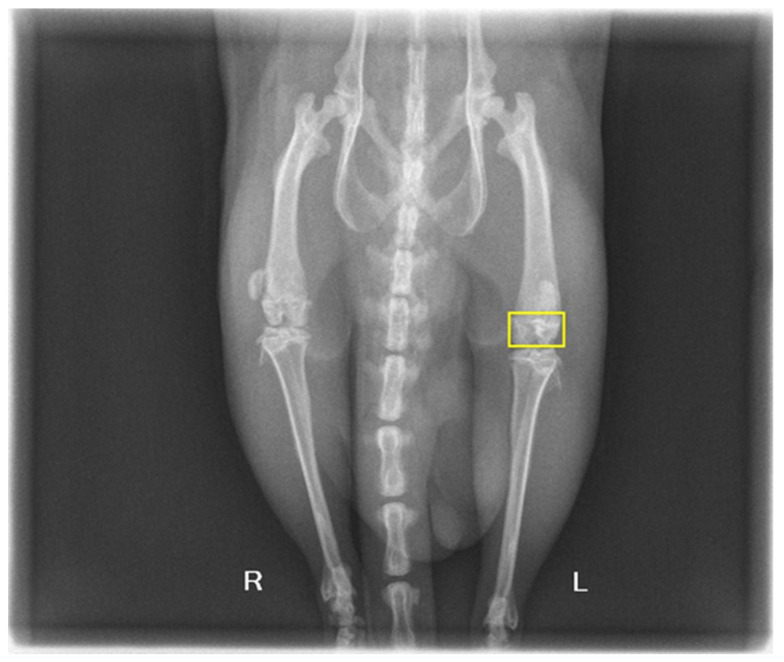
Postoperative X-ray of test subject with area of analysis highlighted.

Rats underwent the surgical procedure as previously described. Subsequently, from the start of the in vivo experiment, half of the animals were monitored for 30 days and the other half for 60 days. The survey included clinical examinations, weight monitoring, hematologic examinations, and postmortem examinations. Observations of gait, posture, and any signs of distress or discomfort were monitored as early signs of the biomaterials’ effect on the experimental organism. The surgical site was observed regularly for signs of infection, dehiscence, or adverse reactions to the materials. From a clinical point of view, the animals responded well, regardless of the substance tested. Thus, upward trends in body weight were observed and no signs of lameness, edema, etc., were observed to indicate any adverse effect of the substances. At the end of the 30 and 60 days, X-ray exams were performed, prior to the animals being humanely euthanized (through an overdose of anesthetic) according to the ethical protocol, as shown in Figure 8.

Radiological non-invasive imaging techniques to monitor the progression of bone regeneration within and around the biomaterials implanted in the rats were employed. Imaging studies were chosen as non-invasive means to monitor the progression of bone regeneration, resorption, and integration with host tissue. The radiographic analysis aimed to measure and quantify the amount of new bone that formed in the areas where the biomaterials were applied. This quantification was critical for assessing the properties of the materials related to the new bone growth on the inside of the implant and on its surface. Another key aspect of the radiographic analysis was to observe and measure the resorption of the biomaterials over time.

**Figure 9:**
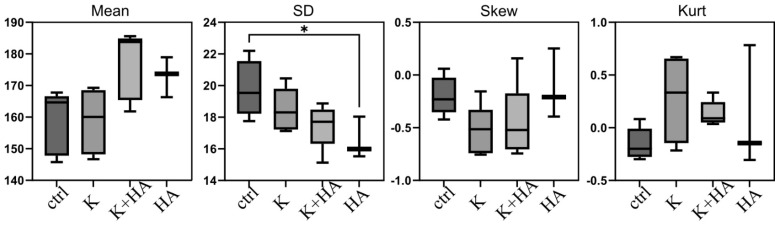
Data for treatment groups were obtained through ImageJ version 1.53 analysis, where the control group (ctrl) is F1, K is F2, HA is F5, and HA + K is F6, as shown in the box plots for Mean, SD, Skew, and Kurt in the provided image. The asterisk (*) specifically in the SD plot signifies a significant difference, highlighting that the variability in the data for the K group (F2) is statistically distinct from the HA group (F5). This finding is important for understanding the consistency and reliability of the results within these groups.

Four parameters related to the pixel intensity distribution in SHG radiological images were monitored and calculated. These parameters were the mean, standard deviation, skewness, and kurtosis (Figure 9). The standard deviation and mean represented frequently used first-order statistical measures, while kurtosis and skewness accounted for the shape of the distribution. The above parameters were assessed in the interest region selected corresponding to the drilled tunnel using the histogram analysis tool comprised in ImageJ. ImageJ is a widely used open-source software with adequate functionalities for performing image processing tasks.

The use of pixel intensity distribution parameters (mean, standard deviation, skewness, and kurtosis) for assessing bone regeneration intensity is well documented. For instance, Kokkinou et al. highlighted the correlation between radiographic texture analysis and biomechanical properties of trabecular bone using these parameters [38]. Similarly, MacKay et al. emphasized the role of higher-order statistics such as skewness and kurtosis in understanding bone texture and regeneration [39].

Data obtained were gathered and processed, cumulated as a single lot. A comparison between different biomaterial groups, treated limb vs. control limb, provided objective data for the research.

The radiological in vivo data obtained suggest that biomaterials may enhance bone density, as indicated by higher mean pixel intensities in the treated groups. This fact is most evident in the case of samples F6 with 0.5% keratin introduced in the Col + HA scaffolds when compared to scaffolds without keratin. A more uniform distribution of pixel intensities, shown in the case of the F6 samples with a decreased standard deviation, hints at a consistent and stable bone structure, likely due to the biomaterials providing a conducive environment for uniform bone growth. Furthermore, the presence of negative skewness and higher positive kurtosis in the treatment groups suggests regions of higher bone density and sharper image features in the group treated with sample F6. These findings imply that the biomaterials not only promote bone growth but also contribute to the formation of precisely defined bone structures, marking effective bone regeneration.

The higher mean pixel intensities for the treatment groups compared to the control group, although not statistically significant, suggest a higher bone density for the F6 group samples.

The standard deviation (SD) of pixel intensities is a measure of the spread or variability within a dataset. Generally, a smaller standard deviation in the pixel intensity distribution might suggest less contrast, indicating a more uniform image. The treatment groups exhibit a decreased standard deviation, indicating a more consistent distribution of pixel values and, consequently, a more uniform bone structure compared to the control group.

Regarding skewness (Skew), all distributions display a negative skew, marked by longer left distribution tails. A decrease in skewness implies a brighter image, indicating regions with higher bone density, as observed in the treatment groups.

All distributions display positive kurtosis (Kurt), nearing zero, with treatment groups showing higher values. This suggests that pixel values are concentrated closer to the mean compared to a normal distribution, with rare extreme deviations contributing minimal variance. The lower standard deviation, linked to higher kurtosis, suggests a tighter spread of pixel values around the mean, indicating sharper features in the image (Figure 9).

Each table below corresponds to a different treatment group and lists parameters including mean, standard deviation (SD), skewness, and kurtosis.

For the exploration of the biomaterial efficacy, image analysis metrics provided critical insights into the tissue responses post-implantation.

The mean pixel intensity within regions of interest (ROIs) quantifies the overall brightness, with higher values typically showing increased bone growth or material deposition. Variability among these pixel intensities, captured by the standard deviation (SD), offers further granularity; a lower SD in treatment groups relative to controls indicates a more uniform response to the biomaterial, potentially signaling consistent bone growth across the treated area.

Furthermore, the skewness and kurtosis of the pixel intensity distribution provide insights into the data’s asymmetry and the sharpness of the distribution peaks. Negative skewness values, when reduced in treatment groups, imply a shift toward more symmetrical or even slightly right-skewed distributions, hinting at a denser and more evenly distributed bone or material density. Higher kurtosis values indicate a more pronounced peak in the distribution, pointing to a more localized and intense area of growth or material presence, thus corroborating the effectiveness of the biomaterials under study.

Table 2 represents the collagen base used across all groups, serving as a control for comparison.

In the control group, utilizing a pure collagen sponge, a slight increase in mean pixel intensity from 161 to 162 was observed over the course from 30 to 60 days, indicating a minor enhancement in bone density or material deposition. The standard deviation remained stable, suggesting consistent variability in bone density across the treated area. Notably, skewness shifted from −0.15 to −0.2, reflecting a trend towards a more symmetrical distribution of pixel intensity, while kurtosis showed a minor increase, suggesting a subtle sharpening of the distribution’s peak.

The next table outlines the statistical distribution of image analysis metrics for group K, which features keratin integrated into the collagen sponge.

The keratin-enhanced collagen group demonstrated a significant increase in mean pixel intensity, from a median of 165 to a third quartile of 181, as shown in Table 3, suggesting robust improvements in bone density or material deposition. The standard deviation maintained a steady value around 19, indicating a consistent variability in the treatment response. Additionally, skewness showed substantial improvement, moving towards a less negative value, which demonstrates a more even distribution of bone growth. Moreover, kurtosis increased, indicative of more pronounced and localized peaks of intense growth at the 60-day mark.

Table 4 displays the distribution of image analysis metrics for group K + HA, which combines keratin and hydroxyapatite with the collagen sponge.

For the composite material group of keratin- and hydroxyapatite-enhanced collagen, the mean pixel intensity rose from 172 at 30 days to 176 at 60 days, highlighting an improvement in the effectiveness of the composite material. The standard deviation saw a slight increase, reflecting a mild rise in the variability of material deposition. Improvements in skewness were noted, moving towards a more balanced material distribution, and kurtosis slightly increased, pointing to more sharply defined peaks in the material’s distribution, suggesting concentrated areas of bone growth.

Table 5 summarizes the distribution of image analysis metrics for group HA, which used hydroxyapatite alone.

In the hydroxyapatite-enhanced collagen group, there was a notable increase in mean pixel intensity from 169 to 177 from 30 to 60 days, signaling enhanced material efficacy. The standard deviation decreased, indicating a reduction in variability and a more uniform response across the treatment area. Skewness also improved, becoming less negative, which may reflect a more symmetrical distribution of the material, and a slight increase in kurtosis was observed, suggesting a sharper peak in the distribution and more localized intense growth.

## 4. Conclusions and Perspectives

The novel biomimetic composites based on collagen, keratin, and hydroxyapatite exhibit desirable morphological and structural properties proved by water absorption, SEM, and FT-IR. The samples have porous structure with pore sizes between 50 and 200 µm, which is ideal for bone scaffolds, and absorbed over 20 g/g water.

Accounting for the biocompatibility and biological performance of the composites, they demonstrated high biocompatibility and promises of good cell viability and proliferation in vitro for samples with maximum 1% keratin. Human bone marrow mesenchymal stem cells (BMSCs) adhered well and thrived on the scaffolds, indicating the material’s potential to facilitate cellular activities essential for bone healing and regeneration, akin to ECM characteristics.

The radiological analysis and subsequent image processing outcomes provide insightful data on the osteoconductive capabilities of the biomaterials tested in rat models. Utilizing non-invasive radiological imaging techniques for monitoring bone regeneration highlights the efficacy and interaction of these biomaterials with host tissue, emphasizing their potential in enhancing bone healing processes.

While the current findings show promising results, especially for samples F5 and F6, further studies are needed to scale the biomaterial for clinical trials and to explore the long-term outcomes of its use in larger animal models and eventually in human subject recipients. The potential customization of this type of composite to suit specific patient needs offers a promising avenue for a more personalized approach in orthopedics.

Future research should focus on refining biomaterial compositions to boost osteoconductive properties and resorption, guided by bone density and structure trends. It is essential to pursue longitudinal studies for a deeper understanding of these materials’ long-term effectiveness and stability. Additionally, integrating advanced imaging techniques like MRI or CT scans with traditional radiology could yield finer details of bone regeneration and material integration induced by the biomaterials. Biomechanical testing will further illuminate the functional outcomes of regeneration, offering a well-rounded view of material performance.

While the current findings show promising results, further studies are needed to scale the biomaterial for clinical trials and to explore the long-term outcomes of its use in larger animal models and eventually in human subject recipients. The potential customization of this type of composite to suit specific patient needs offers a promising avenue for a more personalized approach in orthopedics.

## Figures and Tables

**Table 1:** Composition of biocomposites.

Biocomposite Codes	Collagen *, %	Keratin *, %	Hydroxyapatite, %
F1	1	0	0
F2	1	0.5	0
F3	1	1.0	0
F4	1	1.5	0
F5	1	0	1
F6	1	0.5	1
F7	1	1.0	1
F8	1	1.5	1

* reported as gel amount.

**Table 2:** Control group (ctrl)—collagen sponges (F1).

Parameter	Min Value	1st Quartile	Median	3rd Quartile	Max Value
Mean (30 days)	158	160	161	163	165
SD (30 days)	19	20	21	22	23
Skew (30 days)	−0.3	−0.2	−0.15	−0.1	0.0
Kurt (30 days)	−0.2	−0.1	0.0	0.1	0.2
Mean (60 days)	161	162	163	164	165
SD (60 days)	20	20.5	21	22	23
Skew (60 days)	−0.25	−0.2	−0.15	−0.1	−0.05
Kurt (60 days)	−0.1	0.0	0.05	0.1	0.2

**Table 3:** Group K—collagen sponges with keratin (F2).

Parameter	Min Value	1st Quartile	Median	3rd Quartile	Max Value
Mean (30 days)	145	149	165	181	190
SD (30 days)	16	18	19	21	22
Skew (30 days)	−0.422	−0.3	−0.231	0.05	0.1
Kurt (30 days)	0.082	0.3	0.4	0.5	0.6
Mean (60 days)	180	182	185	190	194
SD (60 days)	18	19	20	21	22
Skew (60 days)	−0.7	−0.6	−0.55	−0.5	−0.4
Kurt (60 days)	0.4	0.5	0.6	0.7	0.9

**Table 4:** Group K + HA—collagen sponges with keratin (K) and hydroxyapatite (HA) (F6).

Parameter	Min Value	1st Quartile	Median	3rd Quartile	Max Value
Mean (30 days)	163	167	172	179	186
SD (30 days)	14	15	17	19	21
Skew (30 days)	−0.632	−0.4	−0.1	0.0	0.2
Kurt (30 days)	−0.59	0.3	0.6	0.7	0.9
Mean (60 days)	174	176	178	186	188
SD (60 days)	15	16	17	19	21
Skew (60 days)	−0.6	−0.5	−0.4	−0.3	−0.1
Kurt (60 days)	0.35	0.5	0.65	0.8	1.0

**Table 5:** Group HA—hydroxyapatite (F5).

Parameter	Min Value	1st Quartile	Median	3rd Quartile	Max Value
Mean (30 days)	157	167	169	177	188
SD (30 days)	16	17	19	22	24
Skew (30 days)	−0.5	−0.4	−0.3	−0.2	−0.1
Kurt (30 days)	0.1	0.2	0.3	0.4	0.5
Mean (60 days)	177	181	183	187	188
SD (60 days)	12	14	16	18	20
Skew (60 days)	−0.5	−0.4	−0.3	−0.2	−0.1
Kurt (60 days)	0.3	0.4	0.5	0.6	0.7

## Data Availability

The raw data supporting the conclusions of this article will be made available by the authors on request.
